# Characterizing spatiotemporal variations of polycyclic aromatic hydrocarbons in Taihu Lake, China

**DOI:** 10.1007/s10661-022-10358-4

**Published:** 2022-08-30

**Authors:** Aili Li, Tim aus der Beek, Jin Zhang, Cora Schmid, Christoph Schüth

**Affiliations:** 1 Institute of Applied Geosciences, Technical University of Darmstadt, 64287 Darmstadt, Germany; 2 IWW Water Centre, Moritzstrasse 26, 45476 Mülheim an der Ruhr, Germany; 3 China Coal Aerial Photogrammetry and Remote Sensing Group Co., Ltd, Xi’an, 710199 China; 4 Yangtze Institute for Conservation and Development, State Key Laboratory of Hydrology-Water Resources and Hydraulic Engineering, Hohai University, Nanjing, 210098 China; 5 Xinjiang Institute of Ecology and Geography, Chinese Academy of Sciences, Urumqi, 830011 China

**Keywords:** Taihu Lake, Water sample, PAH concentration, Temporal variation, Spatial distribution, PAH review

## Abstract

In this study, we analyzed the concentration distributions of 20 polycyclic aromatic hydrocarbons (PAHs) in 41 water samples which were collected from the northern part of Taihu Lake during 4 field campaigns (201511, 201606, 201702 and 201709). The concentrations were determined with GC–MS, and their spatial and seasonal distribution characteristics were interpreted. The results show that 2-ring PAHs present considerably higher concentrations in warm seasons than cold seasons, but the concentrations of the other higher-ring PAHs are rather stable in warm and cold seasons. The distribution patterns of these PAHs might be mainly attributed to ambient temperature effects on the PAH solubility in the water body. Meanwhile, the spatial distributions of the PAH concentrations in cold seasons were rather various in the sampling area, while the distributions in the warm seasons were homogeneous. The different distributions could result from the water recharge from the Yangtze River during cold seasons, which diluted PAH concentrations in the northeastern part of the lake. Furthermore, via literature review on PAH concentrations in water body, PAHs are in a wide range of levels and their patterns are different among the studies, which should be more effected by local factors instead of general PAH properties. The results from this study also present special characteristics of PAHs in Taihu Lake, which exhibit more insight on PAHs existence in water bodies.

**Supplementary Information:**

The online version contains supplementary material available at 10.1007/s10661-022-10358-4.

## Introduction

Taihu Lake catchment is one of the areas with the highest level of urbanization and rural industrialization in China (Shen & Ma, 2005; Yeh et al., 2011), and it has a population of around 40 million accounting for more than 3% of the Chinese population. The lake is located in the center of the Yangtze Delta, covering an area of about 2,340 km^2^ with an average water depth of 1.9 m. It is the third-largest freshwater lake in China and is an important role in flood control and irrigation in the region, also for tourism, shipping, aquaculture and especially as a drinking water source for its neighboring cities (Qin et al., 2007; Tao et al., 2010). There are more than 110 inflows and outflows around the lake. The inflows are mostly located around the northern and western part of the lake, while the outflows are in the eastern and southern part, partly connecting Taihu Lake with the Yangtze River (Qin, 2008). The inflows around Taihu Lake discharge a large amount of industrial effluents, improperly treated municipal sewage, diffused pollutants from agriculture and aquaculture, and accumulated atmospheric depositions into the lake (Li et al., 2021b; Shen et al., 2005; Li et al., 2021a). These discharges attaching complex pollutants gradually lead to serious ecological and water quality deterioration in the lake ecosystems, especially in the northern part of the lake surrounded by more inflows and developed areas (Jiang et al., 2012; Tao et al., 2018; Wang et al., 2003; Wilhelm et al., 2011; Xu et al., 2014). In addition, the water retention time in Taihu Lake is even more than 10 months, which is rather longer than many other lakes and results in more potential for water deterioration (Qin, 2008).

PAHs are ubiquitously distributed organic pollutants in environmental compartments. They consist of two or more aromatic rings and the simplest PAH structure is two-ring naphthalene (naph). The 2-ring and 3-ring PAHs are more volatile and hydrophilic, while with molecular mass increase they become hydrophobic and lipophilic and more resistant to environmental degradation. PAHs can be emitted from both petrogenic and pyrogenic processes, while nowadays, their sources are mainly from pyrolytic processes such as biomass and fossil fuel combustions. US EPA listed 16 PAHs as priority pollutants, and some of PAHs such as benzo[a]pyrene (BaP), dibenzo[a,h]anthracene (DahA), benzo[a]anthracene (BaA) are classified as mutagenic, carcinogenic and genotoxic contaminants (Boffetta et al., 1997; Kim et al., 2013; US EPA, 1993; Arey et al., 2010). It has been reported that bioconcentration factors of hydrophobic organic pollutants including PAHs are positively correlated with their partitioning coefficient K_ow_ (Axelman et al., 1999; Geyer et al., 1984), so heavier and more toxic PAHs have higher potentials to be bioaccumulated in organisms.

Combining the significant roles of Taihu Lake and the ubiquitous source and toxicity of PAHs, it is necessary to investigate the PAH distribution characteristics to assist the mitigation strategies for PAH pollution in surface water. In this study, we analyzed 41 water samples collected from the northern part of Taihu Lake to explain the spatial and seasonal variations of PAH concentrations, and the results were further interpreted to illustrate the effects of ambient conditions and anthropogenic activities on PAH distributions in the water body. Furthermore, previous studies on PAHs in water bodies were reviewed to conclude the general distribution characteristics of PAHs in surface water.

## Materials and methods

**Fig. 1:**
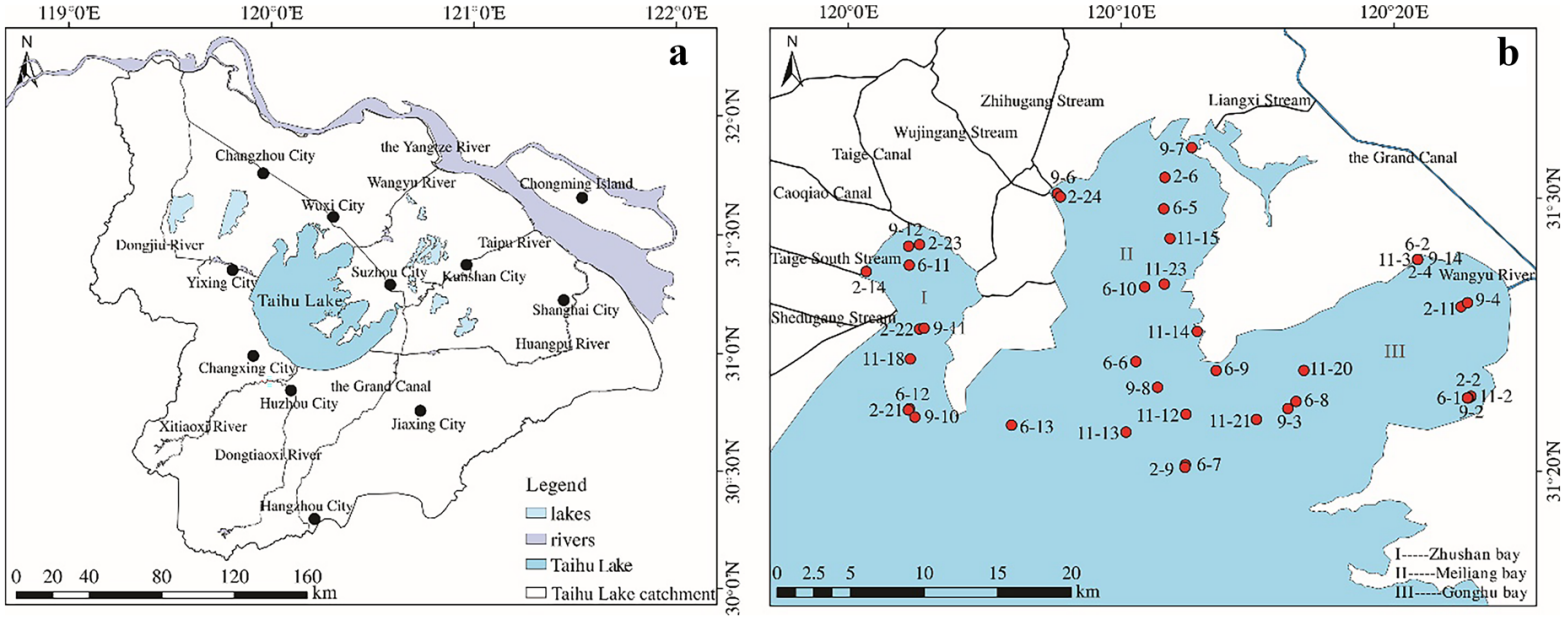
**a** Taihu Lake catchment with main rivers and big cities; **b** water sampling locations (10 samples labeled with 11- were sampled in 2015 Nov.; 11 samples labeled 6- were sampled in 2016 June; 10 samples labeled 2- were sampled in 2017 Feb.; 10 samples labeled 9- were sampled in 2017 Sep.). The coordination of the sampling locations is attached in data supplementary S. Table 1

During 4 field campaigns (2015 Nov., 2016 June, 2017 Feb. and 2017 Sep.), 41 water samples in total were collected from the northern part of Taihu Lake covering Gonghu bay, Meiliang bay and Zhushan bay (Fig. 1). In each location, 2 L of water samples were taken at 0.5 m depth and kept in 2 1-L amber glass bottles for PAH measurement and backup, respectively. After sampling, the samples were immediately transported to the laboratory for PAH extraction.

Around 1 L of each sample was filtered with 0.45-µm pore size filters, then 1 mL (400.2 ng PAH standards) of stock solution was injected as an internal standard to each sample before PAH extraction. PAHs were extracted with ISOLUTE^®^ ENV + 200 mg/3 mL SPE cartridge which was activated with 3 × 3 mL ethyl acetate before PAH loading, and the extraction speed was around 1 L/h. After extraction, the loaded cartridges were dried naturally and then kept in individual sealed polyethylene bags. They were transported to Germany by flight for further PAH analyses.

The cartridges were eluted with 3 × 3 mL of acetone, and the eluates were immediately concentrated to around 1 mL with nitrogen stream. Then 1 µL of the concentrated eluate was injected into a GC–MS (Agilent 7890A/5975C) for PAH concentration measurement. PAHs in GC–MS were carried by helium in pulsed splitless mode and detected in the selected ion monitoring (SIM) mode of the MS. The column is HP- 5MS 5% Phenyl Methyl Silox with 30 m length, 250 µm i.d. and 0.25-µm film thickness. The running time of one complete sequence is 43.333 min. The oven temperature is 75 °C for 3 min and is further programmed to 235 °C at 20 °C/min for 18 min and then to 300 °C at 15 °C/min for 8 min, finally to 320 °C at 10 °C/min.

For quantification, PAH standards (PAH-mix 14, PAH-mix 45 and deuterated PAH-mix 31) were obtained from Dr. Ehrenstorfer Augsburg, Germany. The three standards were diluted together in cyclohexane to four different concentrations for external calibration and response factors calculation. The internal standard (dilution of deuterated PAH-mix 31) with 5 deuterated PAHs was then used to quantify the analytes. All solvents and cleanup chemicals were purchased from Carl Roth GmbH + Co.KG, Germany.

In total, there are 20 PAHs analyzed in this study, including 2-ring: naphthalene (naph), 2-methylnaphthalene (2methylnaph), 1-methylnaphthalene (1methylnaph); 3-ring: acenaphthylene (acenaphthy), acenaphthene (acenaphthe), fluorene, phenanthrene (phen), anthracene (anthra); 4-ring: fluoranthene (fluor), pyrene, benzo[a]anthracene (BaA), chrysene (chry); 5-ring: benzo[b]fluoranthene (BbF), benzo[k]fluoranthene (BkF), benzo[e]pyrene (BeP), benzo[a]pyrene (BaP), perylene, dibenzo[a,h]anthracene (DahA), 6-ring: benzo[g,h,i]perylene (BghiP), indeno[1,2,3-c,d]pyrene (INcdP). The quantification limit for the GC–MS analysis was between 10 and 25 pg of injected mass in a standard, depending on the PAH compound. Since the water volume of each sample is 1 L, this limit corresponds to 10–25 ng/L of individual PAHs in the water samples.

## Results and discussion

### Seasonal variation of PAH concentrations

**Fig. 2:**
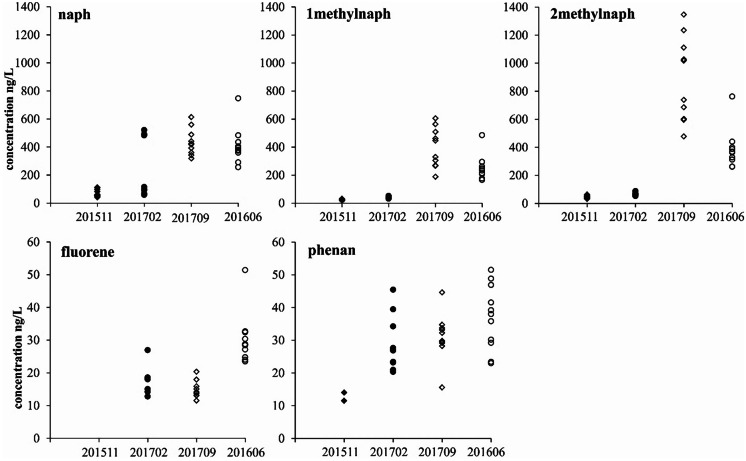
Seasonal variations of PAH concentrations in the lake water (the samples collected in 2015 Nov. are numbered 201511 with labels of filled diamond; the samples collected in 2017 Feb. are numbered 201702 with labels of filled circle; the samples collected in 2017 Sep. are numbered 201709 with labels of unfilled diamond; the samples collected in 2016 June are numbered 201606 with labels of unfilled circle. Samples 201511 and 201702 were collected in cold seasons, 201909 and 201606 were collected in warm seasons)

The detectable PAHs in the water samples are dominated by naph, 2methylnaph and 1methylnaph, and then fluorene and phen are the relatively abundant compounds (The concentration data are attached in the data supplementary S. Table 2). Comparing the four campaigns, the concentrations of naph, 2methylnaph and 1methylnaph in the samples collected in warm seasons of 2016 June (201606) and 2017 Sep. (201709) are up to one order of magnitude higher than that in the samples collected in cold season (2015 Nov. (201511) and 2017 Feb. (201702)) (Fig. 2). Particularly, the concentrations of 2methylnaph in the campaign 201709 are even up to 20 folds higher compared to that in the samples collected in cold season. However, the concentrations of fluorene and phen are rather low and do not show noticeable seasonal variations, and the concentrations of the other PAHs are typically below the quantification limit in both warm and cold seasons.

The water temperature in cold and warm seasons was around 10 °C and 30 °C, respectively. It has been known that the water solubility of some PAHs including naph increase with the increase of temperature (Pérez-tejeda et al., 1990; Whitehouse, 1984), and also, the water partitioning of PAHs is temperature-dependent (Jenkins et al., 1996; Slmonich & Hites, 1994; Tsapakis & Stephanou, 2005). For example, the partitioning of naph and methylnaph from diesel, gasoline and coal tar to water is significantly higher than the partitioning of the other heavier PAHs in ambient temperature, and the temperature effect on partitioning of the other PAHs cannot be detected until 100 ºC or even higher (Lee et al., 1992a, b; Yang et al., 1997). Therefore, the noticeably high concentrations of naph and methylnaph in warm seasons should be mainly attributed to temperature effects. In addition, the concentrations of 1methylnaph and 2methylnaph are similar or higher than that of naph in individual sampling campaign. However, the combustion emission of methylnaph is generally severalfolds lower than that of naph (Herrington et al., 2012; Jenkins et al., 1996; Mcdonald et al., 2000), so it can be suspected that the high concentrations of methylnaph in the warm season samples (201709 and 201606) should result from other sources besides combustion.

### Spatial distribution of PAH concentrations

**Fig. 3:**
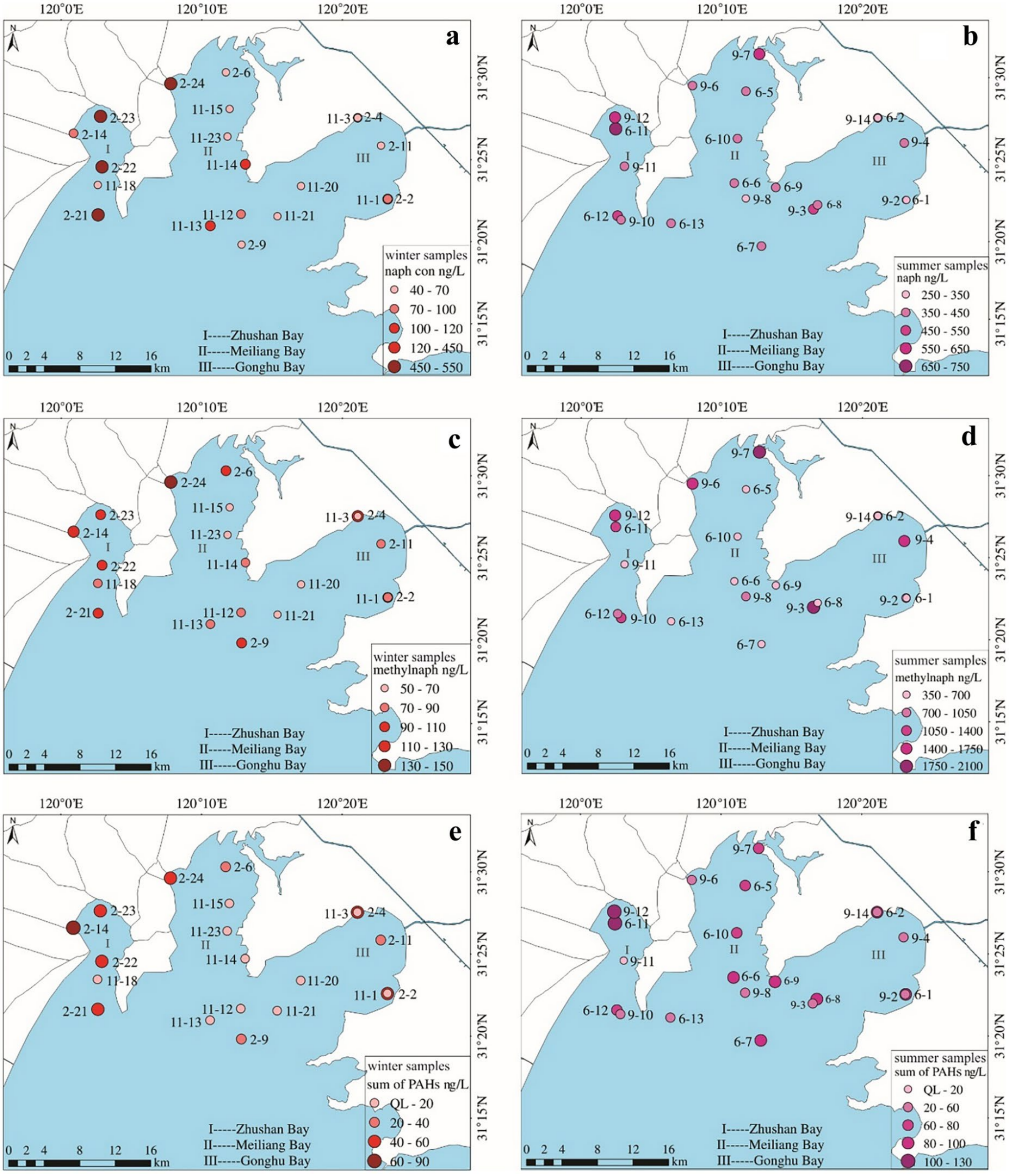
Spatial variation of PAH concentrations in the water body. The figures **a** and **b** are the concentration distributions of naph, c and d are the concentrations of methylnaph (1methylnaph + 2methylnaph), e and f are the sum concentrations of the other PAHs (acenaphthy + acenaphthe + fluorene + phen + anthra + fluor + pyrene). **a**, **c** and **e** are the results from collected in 2015 Nov. and 2017 Feb. in cold seasons, **b**, **d** and **f** are the results from the samples collected in 2016 June and 2017 Sep. in warm seasons. The concentration scale in the figures is different. QL: quantification limit

It is rather consistent in the spatial distributions of the concentrations of naph, methylnaph (1mehtylnaph + 2methylnaph) and the sum of the other PAHs (acenaphthy + acenaphthe + fluorene + phen + anthra + fluor + pyrene) in cold seasons, the higher concentrations are located in Zhushan bay and the lower concentrations are located in Gonghu bay and the eastern part of Meiliang bay (Fig. 3a, c, e). The spatial distributions of the concentrations of naph and the other PAHs in warm season, however, are somewhat homogeneous (Fig. 3b, f). However, the spatial distribution of methylnaph concentrations in warm season does not show specific spatial patterns, and they present marked differences between the two campaigns, that is, significantly high concentrations in samples 201709 and relatively low concentrations in samples 201606 (Fig. 3d). The inconsistent distributions of methylnaph with the other PAHs further support the suspicion that there are additional origins of methylnaph particularly in the sampling period of Sep. 2017.

Generally, from Nov. to the following April is the dry season in this area and the water level in the lake decreases significantly, while there is rather more precipitation in the other months in the catchment, especially monsoon occurs almost in every summer resulting in heavy precipitation and flood (Chen et al., 2001; Hu et al., 2021; Tao et al., 2012; Xu et al., 2007). The general inflows are located around the northern and the southwestern part of the lake and the outflows are around the eastern and southeastern part of the lake (Qin, 2008), which result in the water flow direction in the lake typically from the western part to the southeastern part. The water level in Taihu Lake is somehow adjusted by water recharge from the Yangtze River through the Wangyu River connecting the northeastern part of Taihu Lake (Gonghu bay) (Fig. 1a) (Zhai et al., 2010). In dry season, there is less water input from the inflow rivers in the northern and western part of the lake, so the water recharge from the Yangtze River to the northeastern part of Taihu Lake can somewhat change the water flow directions in the northern part of the lake. The time allocation of the water recharge mainly depends on the lake water level, which can be obtained in the documents of monthly and yearly water regime downloaded from Taihu Basin Authority of Ministry of Water Resources (http://www.tba.gov.cn/channels/48.html).

The samples 201511 and 201702 were collected in dry seasons with water recharge from the Yangtze River, but the samples 201606 and 201709 were collected during rainy seasons with more water inflow from the rivers around the northern and northwestern part of the lake and also more water discharge to the Yangtze River. The PAH concentration distributions in samples 201511 and 201702 present that higher concentrations were located in Zhushan bay and in the western of Meiliang bay close to the inflow river outlets, and lower concentrations were located in the eastern area of Meiliang bay and Gonghu bay (Fig. 3a, c, e). These spatial distributions suggest that PAH abundance in the Yangtze River was lower than that in the northern part of Taihu Lake, and PAH concentrations in Gonghu bay and the eastern Meiliang were diluted by the water recharge from the Yangtze River. However, the concentration distributions in samples 201606 and 201709 were rather homogeneous, which can be attributed to that in rainy season, there is more water input from the other inflow rivers and high water dynamics in the lake homogenize PAH concentration distributions in the water body.

### Review other studies on PAHs in water

As this research is focused on dissolved PAHs in the lake water, the comparison and discussion from other publications are also based on dissolved PAHs in surface water. Most researches only measured the 16 PAHs listed by US EPA, and few researches also measured other PAHs such as 1mehtylnaph and 2methylnaph (Guo et al., 2011; Tongo et al., 2017). There are variable PAH patterns and orders of magnitude variations in their concentration levels in different studies. The detected PAHs are generally dominated by 2-ring and 3-ring PAHs, especially 2-ring naph and 3-ring phen (Bhutto et al., 2021; Li et al., 2006, 2010; Oyo-Ita et al., 2021; Sun et al., 2009), which is similar with the PAH patterns in this study. Meanwhile, the concentrations of 3-ring acenaphthy and acenaphthe in some studies are found as high as the concentration of naph (Kabziński et al., 2002; Tongo et al., 2017; Sarria-villa et al., 2016; Kafilzadeh, 2015; Ambade et al., 2021). The 4-ring fluor and pyrene are generally the heaviest PAHs detected in water samples (Li et al., 2006, 2010; Sun et al., 2009), but in some researches 5-ring and 6-ring PAHs also show relatively high concentrations (Dhananjayan et al., 2012; Edori & Odoemelam, 2022; Guo et al., 2011). However, markedly high concentrations of 1methylnaph and 2methylnaph as that found in the current study have not been investigated and/or discovered in previous studies. The concentrations of individual PAHs range from several ng/L to thousands ng/L in different studies, and the variations mostly occur in the 2-ring and 3-ring PAHs (Dhananjayan et al., 2012; Kafilzadeh, 2015; Patrolecco et al., 2010; Sarria-villa et al., 2016; Tongo et al., 2017; Yang et al., 2013).

Seasonal variations of PAH concentrations in water are also not consistent: higher PAH concentrations in warm season than in cold season (Deng et al., 2006; Matsunaka et al., 2022; Montuori et al., 2016; Qin et al., 2014), which is similar to this study; higher concentrations in cold season than in warm season (Guigue et al., 2014; Liu et al., 2016a, b; Mzoughi & Chouba, 2011); no obvious differences between cold and warm seasons (Guigue et al., 2011; Kafilzadeh, 2015; Liu et al., 2016a, b). In addition, it was also found that naph was the dominant in cold seasons, but the 3-ring PAHs were the dominant in warm seasons (Li et al., 2015a, b). Combing the seasonal variations of PAH concentrations, it indicates that PAH distributions in water are specifically related to local situation rather than temperature-affected partitioning. Therefore, even though there are significant amount of researches on PAHs in water phase, it is difficult to conclude general characteristics of PAH distribution and existence in water bodies.

## Conclusion

In Taihu Lake water, there are higher concentrations of PAHs in warm seasons than cold seasons, which is mainly attributed to temperature effects rather than the other factors. In cold seasons, the higher PAH concentrations were located in the northwestern part of the lake, while the relatively concentrations were distributed in the northeastern part. This distribution patterns mainly resulted from the water recharge from the Yangtze River through the outlet located in the northeastern part of the lake. In warm seasons, the PAH concentration distributions were more homogeneous, which is attributed to more precipitation and other inflows increasing the water dynamics and homogenizing PAH concentration distributions. According to the literature review on PAH concentrations in surface water bodies, different studies present different PAH patterns and seasonal variations, suggesting that specific local situations have more effects on PAH existence than its general properties.

## Supplementary Information

Below is the link to the electronic supplementary material.

Supplementary file1 (DOCX 22 KB)
