# Supplementary material for: Characterizing spatiotemporal variations of polycyclic aromatic hydrocarbons in Taihu Lake, China
Source: Environ Monit Assess. 2022 Aug 30;194(10):713. doi: 10.1007/s10661-022-10358-4 (PMC9427900; doi:10.1007/s10661-022-10358-4)
Supplement: Supplementary file 1 — Supplementary file1 (DOCX 22 KB) [file 10661_2022_10358_MOESM1_ESM.docx]

**Data Supplementary**

**Characterizing spatiotemporal variations of polycyclic aromatic hydrocarbons in Taihu Lake, China**

*Aili Li^1,3*^, Tim aus der Beek^2^, Jin Zhang^4,5^, Cora Schmid^2^, Christoph Schüth^1,2^*

^1^ Institute of Applied Geosciences, Technical University of Darmstadt, Darmstadt, 64287, Germany

^2^ IWW Water Centre, Moritzstrasse 26, 45476 Mülheim an der Ruhr, Germany

3 China Coal Aerial Photogrammetry and Remote Sensing Group Co., Ltd., 710199, Xi’an, China

^4^ Yangtze Institute for Conservation and Development, State Key Laboratory of Hydrology-Water Resources and Hydraulic Engineering, Hohai University, 210098 Nanjing, China

^5^ Xinjiang Institute of Ecology and Geography, Chinese Academy of Sciences, 830011 Urumqi, China

Corresponding author: Aili Li [lial11@lzu.edu.cn](mailto:lial11@lzu.edu.cn)

ORCID: 0000-0002-9464-8726

**S. Table 1** Sampling time and location

| **location** | **sampling time** | **N** | **E** | **location** | **sampling time** | **N** | | **E** | |
| --- | --- | --- | --- | --- | --- | --- | --- | --- | --- |
| 11-2 | 2015-11 | 31.3793 | 120.3805 | 2-2 | 2017-02 | 31.3753 | | 120.3889 | |
| 11-3 | 2015-11 | 31.4627 | 120.3479 | 2-4 | 2017-02 | 31.4627 | | 120.3479 | |
| 11-12 | 2015-11 | 31.3683 | 120.2064 | 2-6 | 2017-02 | 31.5128 | | 120.1935 | |
| 11-13 | 2015-11 | 31.3574 | 120.1697 | 2-9 | 2017-02 | 31.3374 | | 120.2060 | |
| 11-14 | 2015-11 | 31.4189 | 120.2133 | 2-11 | 2017-02 | 31.4337 | | 120.3745 | |
| 11-15 | 2015-11 | 31.4755 | 120.1966 | 2-14 | 2017-02 | 31.4552 | | 120.0109 | |
| 11-18 | 2015-11 | 31.4021 | 120.0379 | 2-21 | 2017-02 | 31.3717 | | 120.0373 | |
| 11-20 | 2015-11 | 31.3951 | 120.2784 | 2-22 | 2017-02 | 31.4204 | | 120.0436 | |
| 11-21 | 2015-11 | 31.3651 | 120.2494 | 2-23 | 2017-02 | 31.4718 | | 120.0435 | |
| 11-23 | 2015-11 | 31.4476 | 120.1931 | 2-24 | 2017-02 | 31.5029 | | 120.1277 | |
| 6-1 | 2016-06 | 31.0033 | 120.4681 | 9-2 | 2017-09 | 31.3753 | | 120.3889 | |
| 6-2 | 2016-06 | 31.4626 | 120.3480 | 9-3 | 2017-09 | 31.3718 | | 120.2686 | |
| 6-5 | 2016-06 | 31.4936 | 120.1928 | 9-4 | 2017-09 | 31.4362 | | 120.3784 | |
| 6-6 | 2016-06 | 31.4006 | 120.1758 | 9-6 | 2017-09 | 31.5009 | | 120.1296 | |
| 6-7 | 2016-06 | 31.3358 | 120.2058 | 9-7 | 2017-09 | 31.5310 | | 120.2100 | |
| 6-8 | 2016-06 | 31.3761 | 120.3761 | 9-8 | 2017-09 | 31.3847 | | 120.1890 | |
| 6-9 | 2016-06 | 31.3950 | 120.2247 | 9-10 | 2017-09 | 31.3666 | | 120.0407 | |
| 6-10 | 2016-06 | 31.4458 | 120.1811 | 9-11 | 2017-09 | 31.4209 | | 120.0464 | |
| 6-11 | 2016-06 | 31.4592 | 120.0372 | 9-12 | 2017-09 | 31.4708 | | 120.0368 | |
| 6-12 | 2016-06 | 31.3708 | 120.0367 | 9-14 | 2017-09 | 31.4626 | | 120.3480 | |
| 6-13 | 2016-06 | 31.3617 | 120.0997 |  |  |  |  | |  |

Geographic Coordinate System: GCS_WGS_1984

| **S. Table 2** PAH concentration in the water samples (ng/L) | | | | | | | | | | |  |
| --- | --- | --- | --- | --- | --- | --- | --- | --- | --- | --- | --- |
| location | naph | 2methylnaph | 1methylnaph | acenaphthy | acenaphthe | fluorene | phen | anthra | fluor | pyrene | |
| 11-2 | 81.84 | 46.10 | 27.18 | ND | ND | ND | 13.99 | ND | ND | ND | |
| 11-3 | 55.53 | 48.50 | 18.83 | ND | ND | ND | ND | ND | ND | ND | |
| 11-12 | 96.07 | 45.58 | 25.59 | ND | ND | ND | ND | ND | ND | ND | |
| 11-13 | 112.47 | 56.31 | 31.18 | ND | ND | ND | 11.51 | ND | ND | ND | |
| 11-14 | 109.31 | 50.27 | 29.18 | ND | ND | ND | ND | ND | ND | ND | |
| 11-15 | 48.25 | 32.29 | 20.33 | ND | ND | ND | ND | ND | ND | ND | |
| 11-18 | 60.38 | 62.98 | 21.03 | ND | ND | ND | ND | ND | ND | ND | |
| 11-20 | 41.62 | 42.22 | 19.16 | ND | ND | ND | ND | ND | ND | ND | |
| 11-21 | 51.24 | 34.18 | 19.57 | ND | ND | ND | ND | ND | ND | ND | |
| 11-23 | 57.93 | 35.78 | 21.16 | ND | ND | ND | ND | ND | ND | ND | |
| 6-1 | 255.34 | 262.39 | 165.85 | ND | ND | 23.98 | 46.86 | ND | ND | ND | |
| 6-2 | 289.67 | 261.37 | 176.13 | ND | ND | 32.64 | 51.47 | ND | ND | ND | |
| 6-5 | 379.00 | 362.97 | 233.63 | ND | 10.67 | 28.42 | 30.16 | ND | ND | ND | |
| 6-6 | 396.71 | 367.30 | 233.80 | ND | 12.42 | 28.75 | 48.80 | ND | ND | ND | |
| 6-7 | 393.13 | 386.71 | 247.58 | ND | 12.98 | 27.12 | 29.20 | ND | ND | ND | |
| 6-8 | 403.23 | 398.67 | 245.01 | ND | 13.18 | 30.36 | 37.98 | ND | ND | ND | |
| 6-9 | 434.72 | 387.20 | 260.57 | 10.14 | 15.53 | 32.37 | 35.78 | ND | ND | ND | |
| 6-10 | 373.41 | 311.60 | 208.16 | ND | 12.05 | 24.74 | 39.18 | ND | ND | ND | |
| 6-11 | 746.66 | 761.80 | 484.70 | 11.01 | 21.69 | 51.40 | 41.50 | ND | ND | ND | |
| 6-12 | 483.07 | 439.36 | 294.71 | ND | 10.12 | 28.74 | 23.39 | ND | ND | ND | |
| 6-13 | 358.75 | 327.86 | 213.10 | ND | ND | 23.47 | 22.96 | ND | ND | ND | |

| **S. Table 2** PAH concentration in the water samples (ng/L) | | | | | | | | | | |  |
| --- | --- | --- | --- | --- | --- | --- | --- | --- | --- | --- | --- |
| location | naph | 2methylnaph | 1methylnaph | acenaphthy | acenaphthe | fluorene | phen | anthra | fluor | pyrene | |
| 2-2 | 118.88 | 69.36 | 38.94 | ND | ND | 12.16 | 33.27 | ND | ND | ND | |
| 2-4 | 97.85 | 72.39 | 41.89 | ND | ND | 15.01 | 39.45 | ND | ND | ND | |
| 2-6 | 62.39 | 56.89 | 33.38 | ND | ND | 12.74 | 20.90 | ND | ND | ND | |
| 2-9 | 66.70 | 66.57 | 36.49 | ND | ND | 14.17 | 23.37 | ND | ND | ND | |
| 2-11 | 58.39 | 51.87 | 34.75 | ND | ND | ND | 20.35 | ND | ND | ND | |
| 2-14 | 91.26 | 62.19 | 51.58 | ND | 10.39 | 26.92 | 34.20 | 13.10 | ND | ND | |
| 2-21 | 487.00 | 62.40 | 32.32 | ND | ND | 18.12 | 27.60 | ND | ND | ND | |
| 2-22 | 483.15 | 61.92 | 32.46 | ND | ND | 18.00 | 23.19 | ND | ND | ND | |
| 2-23 | 520.48 | 62.17 | 33.71 | ND | ND | 18.50 | 26.85 | ND | ND | ND | |
| 2-24 | 491.00 | 87.69 | 44.51 | ND | ND | 18.55 | 27.14 | ND | ND | ND | |
| 9-2 | 318.31 | 595.93 | 269.95 | ND | ND | 11.48 | 29.52 | ND | ND | ND | |
| 9-3 | 488.26 | 1235.23 | 563.71 | ND | ND | 15.04 | 29.81 | ND | ND | ND | |
| 9-4 | 423.70 | 1026.78 | 460.95 | ND | ND | 13.03 | 33.69 | ND | ND | ND | |
| 9-6 | 440.91 | 1111.41 | 508.97 | ND | ND | 15.90 | 34.73 | ND | ND | ND | |
| 9-7 | 559.06 | 1347.05 | 605.13 | ND | 10.27 | 17.93 | 32.23 | ND | ND | ND | |
| 9-8 | 341.20 | 601.94 | 265.84 | ND | ND | 13.15 | 29.14 | ND | ND | ND | |
| 9-10 | 392.01 | 738.69 | 329.47 | ND | ND | 13.79 | 33.17 | ND | ND | ND | |
| 9-11 | 359.56 | 477.20 | 188.30 | ND | ND | ND | 15.55 | ND | ND | ND | |
| 9-12 | 612.86 | 1018.76 | 446.67 | ND | 14.08 | 20.34 | 44.59 | ND | 11.88 | 14.47 | |
| 9-14 | 417.68 | 685.01 | 304.72 | ND | ND | 14.14 | 28.21 | ND | ND | ND | |
